# Association between sexual behaviour and head and neck cancer in the French West Indies: a case-control study based on an Afro-Caribbean population

**DOI:** 10.1186/s12885-023-10870-x

**Published:** 2023-05-05

**Authors:** Aviane Auguste, Stanie Gaete, Léah Michineau, Cécile Herrmann-Storck, Clarisse Joachim, Suzy Duflo, Jacqueline Deloumeaux, Danièle Luce

**Affiliations:** 1 Univ Rennes, Inserm, EHESP, Irset (Institut de recherche en santé, environnement et travail) -UMR_S 1085, Pointe-à-Pitre, F-97100 France; 2 Biological Resources Center Karubiotec™ BRIF n° KARUBIOTEC-GUA00971, University Hospital of Guadeloupe, Pointe-à-Pitre, Guadeloupe; 3 Laboratory of Microbiology, University Hospital of Guadeloupe, Pointe-à-Pitre, Guadeloupe; 4 Registre des cancers, Martinique Cancer Registry, UF 1441 Pôle de Cancérologie Hématologie Urologie Pathologie, University Hospital of Martinique, Fort-de-France, Martinique, France; 5 Department of Oto-Rhino-Laryngology and Head and Neck Surgery, University Hospital of Guadeloupe, Pointe-à-Pitre, Guadeloupe, France; 6 General Cancer Registry of Guadeloupe, University Hospital of Guadeloupe, Pointe-à-Pitre, Guadeloupe, France

**Keywords:** Head and neck cancer, Sexual behaviour, Condoms, Papillomavirus Infections, Sexually transmitted infection, Caribbean

## Abstract

**Background:**

Worldwide, a significant proportion of head and neck cancers is attributed to the Human papillomavirus (HPV). It is imperative that we acquire a solid understanding of the natural history of this virus in head and neck squamous cell carcinoma (HNSCC) development. Our objective was to investigate the role of sexual behaviour in the occurrence of HNSCC in the French West Indies. Additionally, we evaluated the association of high risk of HPV (Hr-HPV) with sexual behaviour in risk of cancer.

**Methods:**

We conducted a population-based case-control study (145 cases and 405 controls). We used logistic regression models to estimate adjusted odds-ratios (OR), and their 95% confidence intervals (CI).

**Results:**

Compared to persons who never practiced oral sex, those who practiced at least occasionally had a lower HNSCC risk. First sexual intercourse after the age of 18 year was associated with a 50% reduction of HNSCC risk, compared to those who began before 15 years. HNSCC risk was significantly reduced by 60% among persons who used condoms at least occasionally. The associations for ever condom use and oral sex were accentuated following the adjustment for high-risk HPV (Hr-HPV). Oral Hr-HPV was associated with several sexual behaviour variables among HNSCC cases. However, none of these variables were significantly associated with oral HPV infections in the population controls.

**Conclusion:**

First intercourse after 18 years, short time interval since last intercourse and ever condom use were inversely associated with HNSCC independently of oral Hr-HPV infection. Sources of transmission other than sexual contact and the interaction between HPV and HIV could also play a role in HNSCC etiology.

**Supplementary Information:**

The online version contains supplementary material available at 10.1186/s12885-023-10870-x.

## Introduction

Head and neck cancer is a public health concern across the world, counting 700,000 new cases every year [1]. This incidence is particularly elevated in Guadeloupe and Martinique which have one of the highest rates among men in Latin America and the Caribbean [1, 2] despite low prevalence of tobacco and alcohol [3]. Oral HPV infection is a prominent risk factor of head and neck cancer.

The incidence of HPV-positive head and neck squamous cell carcinoma (HNSCC) has increased considerably in the past decade [4]. Consequently, to implement effective prevention we require a solid understanding of the natural history of HPV in HNSCC. In spite of the biological similarities to cervical cancer, the etiological pathway in regards to sexual behaviour and oral HPV infection is less clear in HNSCC [5, 6]. Sexual behaviour, including oral sex and other at-risk and promiscuous behaviour have been consistently regarded as plausible drivers of oral HPV infection which in turn provoke HNSCC [7, 8]. We have previously demonstrated a significant association with oral high-risk HPV (Hr-HPV) [9]. Oral Hr-HPV was associated with a two-fold increase in the risk of HNSCC.

However, results from previous studies on sexual behaviour and HNSCC are sometimes conflicting, and few data exist on this topic in populations of African descent [8]. In the current paper we proposed an analysis investigating the association between sexual behaviour and the occurrence of HNSCC in the French West Indies (FWI), and the role of oral Hr-HPV in this association.

## Method

### Study population, data and specimen collection

We conducted a population-based case-control study in Guadeloupe and Martinique. The study is an extension of a large nationwide case-control study, the ICARE study, which has already been conducted in ten French regions covered by a cancer registry [10]. The study in the FWI used the same protocol and questionnaire, described in detail elsewhere [10, 11], with some adaptations to the local context. Eligible cases were patients residing in the FWI, suffering from a primary, malignant tumour of the oral cavity, pharynx, sinonasal cavities and larynx of any histological type, aged between 18 and 75 years old at diagnosis, newly diagnosed and histologically confirmed between April 1, 2013 and June 30, 2016. Incident cases were identified in collaboration with the population-based cancer registries of Guadeloupe and Martinique which use standardized procedures for the recording of all cancer cases in each of these regions. Our study benefitted from the diverse data sources from the registries in order to flag new cases diagnosed during the study period.

The control group was selected from the general population by random digit dialling, using incidence density sampling method. Controls were frequency matched to the cases by sex, age and region. Additional stratification was used to achieve a distribution by socioeconomic status among the controls comparable to that of the general population.

Cases and controls were interviewed face-to-face with a questionnaire including sociodemographic characteristics, lifetime tobacco and alcohol consumption and sexual behaviour. Participants were also asked to provide a saliva sample, using the Oragene® OG-500 kit (DNA Genotek).

Among the 235 eligible cases, 170 (72.3%) agreed to participate and were interviewed. Among the 497 eligible controls, 405 (81.5%) answered the questionnaire. Among cases and controls with interview data, 114 cases (67.1%) and 311 controls (76.8%) provided a saliva sample. Each subject included in the study gave a written and informed consent. The study was approved by the Institutional Review Board of the French National Institute of Health and Medical Research (IRB INSERM n°01–036) and by the French Data Protection Authority (n° DR-2015-2027).

### HPV detection and genotyping

HPV detection in tumours is informative on the involvement of HPV in cancer development. Given the participation of healthy individuals, we resorted to detection of HPV-integrated DNA from saliva samples for both cases and controls. Compared to HPV detection from tumour tissue, the method using saliva still has good specificity [12]. We performed HPV detection using the INNO-LiPA ® kit, according to the manufacturer’s instructions (INNO-LiPA HPV Genotyping *Extra*; Innogenetics, Ghent, Belgium). The INNO-LiPA HPV genotyping assay allows the detection of the following genotypes: HPV16, HPV18, HPV31, HPV33, HPV35, HPV39, HPV45, HPV51, HPV52, HPV56, HPV58, HPV59, HPV68 (High-risk), HPV26, HPV53, HPV66, HPV70, HPV73, HPV82 (Probable high-risk), HPV06, HPV11, HPV40, HPV42, HPV43, HPV44, HPV54, HPV61, HPV81 (Low-risk), HPV62, HPV67, HPV83, HPV89 (Other). The full details on the method for HPV detection has been described elsewhere [11].

### Collection of data on sexual behaviour

Lifetime sexual behavior was ascertained during the face-to-face interviews. The questionnaire included questions pertaining to the number of lifetime sex partners, sexual orientation and whether or not the last sexual intercourse took place in the last 6 months prior to the interview. Participants were asked if they ever performed certain sexual practices and the frequency at which they did them. These sexual practices were condom use, oral sex practice, whether or not the participants had ever received sperm in their mouth. The age at which these acts were last practiced was also noted. Information on having multiple partners, sexual intercourse in exchange for money and sexually transmitted infections (STI) were also collected. Oral sex was defined as the contact between the participant’s mouth and their partner’s genitalia. Having multiple partners was defined as having several sexual partners during the same period. When the frequency of an activity was requested, four responses were possible: just once, sometimes, often, always or almost always. We considered “occasional” behaviour to be “once” or “sometimes”.

### Sampling

We restricted the current analysis to squamous cell carcinomas of the oral cavity (International Classification of Diseases 10th revision codes C00.3-C00.9, C02.0-C02.3, C03.0, C03.1, C03.9, C04.1, C04.8, C04.9, C05.0, C06.0-C06.2, C06.8 and C06.9, n = 35), the oropharynx (ICD-10 codes C01.9, C02.4, C05.1, C05.2, C09, C10, C 14.2, n = 58), the hypopharynx (ICD-10 codes C12- C13, n = 19) and the larynx (ICD-10 codes C32, n = 32). Our analysis included 145 cases and 405 controls.

### Statistical analysis

The association between sexual behaviour variables and the occurrence of HNSCC, and oral Hr-HPV infection was assessed by estimating odds ratios (ORs) and their 95% confidence intervals (CIs), using logistic regression models. Regression analyses were adjusted for age, sex and region, tobacco, alcohol and education level. Tobacco was accounted as one variable combining the quantity (the average number of cigarettes per day over one’s lifetime) and the duration of lifetime smoking.

In order to assess the role of Hr-HPV as a mediator in the relationship between sexual behaviour and HNSCC we performed logistic regressions with Hr-HPV as a covariate as well as reproducing the initial analyses by Hr-HPV subgroups (Hr-HPV-negative and Hr-HPV-positive). Oral Hr-HPV status was assessed as Hr-HPV-positive versus Hr-HPV-negative, the latter category grouping HPV-negative and non-high-risk-HPV genotypes. The association of sexual behaviour and head and neck cancer is supposedly mediated by Hr-HPV. We wanted to assess whether the effect of the sexual behaviour could be explained at least partially by Hr-HPV (hypothesised mediator) [13]. Assuming that Hr-HPV is on the causal pathway to head and neck cancer, we considered HPV as a mediator when the association between sexual behaviour and HNSCC dissipated in the Hr-HPV-positive subgroup. Hr-HPV was also regarded as a mediator when the adjustment for Hr-HPV resulted in the loss of the initial significant association.

We also studied the associations between sexual behaviour and oral Hr-HPV infection separately among population controls and HNSCC cases.

## Results

### Characteristics of the study population

The 55–64 years age group that was most represented in our sample (cases: 42%, controls: 32%). Majority of the study participants were men (cases: 88%, controls:76%). There were more participants from Guadeloupe than Martinique.

### Sexual behaviour and head and neck cancer

Last intercourse beyond 6 months preceding the interview was positively associated with the occurrence of HNSCC (Table 1). Having sexual intercourse after the age of 18 year was associated with a 60% risk reduction, compared to those who began before 15 years. Similarly, HNSCC risk was significantly reduced by 50% among persons who used condoms at least occasionally (once, sometimes). After adjustment for main confounding variables, condom users were twice as likely to have engaged in sexual intercourse in the 6 months prior to their interview compared to never condom users (OR = 2.52, 95% CI = 1.51–4.18) (Data not shown). Receiving money for performing sexual intercourse was uncommon in our study, there were only 6 controls who responded “yes”, and represented 1% of the general population. Oral sex, lifetime sex partners, sexual orientation, paying for sex, and having multiple partners were not associated with HNSCC.

**Table 1:** Association between sexual behaviour and HNSCC

	Case	Control		
	n (col%)	n (col%)	OR^a^	95% CI
**Age at first sexual intercourse (years)**				
< 15	39 (33.9)	71 (17.9)	1	ref
15–18	61 (53.0)	217 (54.8)	0.58	(0.31–1.06)
> 18	15 (13.0)	108 (27.3)	0.41	(0.19–0.91)
Missing	30	9		
**Time since last intercourse (months)**				
≤ 6	61 (56.5)	309 (79.2)	1	ref
> 6	47 (43.5)	81 (20.8)	2.37	(1.33–4.22)
Missing	37	15		
**Number of lifetime partners**				
1 to 5	47 (43.5)	177 (45.5)	1	ref
6 to 20	33 (30.6)	154 (39.6)	0.56	(0.29–1.07)
> 20	28 (25.9)	58 (14.9)	0.87	(0.41–1.85)
Missing	37	16		
**Sexual orientation**				
Heterosexual	89 (80.9)	345 (88.7)	1	ref
Non-Heterosexual	21 (19.1)	44 (11.3)	1.77	(0.86–3.67)
Missing	35	16		
**Condom use**				
Ever	61 (57.0)	303 (77.3)	0.51	(0.28–0.93)
Never	46 (43.0)	89 (22.7)	1	ref
Missing	38	13		
**Condom use, frequency**				
Never	46 (43.0)	89 (22.6)	1	ref
Once, sometimes	40 (37.4)	183 (46.5)	0.51	(0.27–0.97)
Often, always or almost always	21 (19.6)	122 (31.0)	0.51	(0.24–1.08)
Missing	38	11		
**Oral sex**				
Ever	69 (63.3)	288 (72.4)	0.76	(0.42–1.38)
Never	40 (36.7)	106 (26.6)	1	ref
Missing	36	11		
**Oral sex, frequency**				
Never	40 (37.0)	106 (27.4)	1	ref
Once, sometimes	39 (36.1)	201 (51.9)	0.67	(0.35–1.27)
Often, always or almost always	29 (26.9)	80 (20.7)	0.88	(0.41–1.89)
Missing	37	18		
**Received sperm in mouth**				
Never oral sex, never sperm, just once	66 (90.4)	233 (91.4)	1	ref
Sometimes, often, always or almost always	7 (9.6)	22 (8.6)	1.81	(0.56–5.92)
Missing	72	150		
**Paid for sex**				
Ever	27 (24.1)	95 (23.9)	1	ref
Never	85 (75.9)	302 (76.1)	1.54	(0.82–2.86)
Missing	33	8		
**Sexually transmitted infection, frequency**				
Never	75 (68.8)	248 (63.1)	1	ref
Once	11 (10.1)	60 (15.3)	0.51	(0.19–1.18)
More than once	23 (21.1)	85 (21.6)	0.88	(0.44–1.74)
Missing	36	12		
**Recent multiple partners**				
Never multiple partners	68 (61.3)	244 (61.8)	1	ref-
≤ 5 years	12 (10.8)	42 (10.3)	0.93	(0.39–2.22)
> 5 years	31 (27.9)	109 (27.6)	0.81	(0.40–1.48)
Missing	34	10		

^a^OR adjusted for age, sex, region, cigarette quantity and duration combined, alcohol quantity and level of education

We were interested in the role of Hr-HPV in the mediation of the associations between certain sexual behaviours and HNSCC from our analyses (Table 2). The associations observed for age at first intercourse were unchanged after adjusting for Oral Hr-HPV. Stratification was not possible for this variable because of too few subjects in the Hr-HPV-positive group. Nonetheless, the HPV-adjusted ORs for age at first intercourse were similar to that of the Hr-HPV-negative group. The associations for time since last intercourse, ever condom use and oral sex were accentuated following the adjustment for Hr-HPV. In addition, the significant associations between condom use, time since last intercourse and HNSCC appeared only in Hr-HPV-negative HNSCC whereas the association with oral sex remained non-significant regardless of HPV status.

**Table 2:** Association between age at first intercourse, time since last intercourse, condom use, oral sex and HNSCC accounting for Hr-HPV.

	Conf	Conf + Hr-HPV		Hr-HPV-	Hr-HPV+
	**OR**^**a**^ (95% CI)	**OR**^**b**^ (95% CI)		**OR**^**a**^ (95% CI)	**OR**^**a**^ (95% CI)
**Age at first sexual intercourse**					
< 15	1 (ref)	1 (ref)		1 (ref)	1 (ref)
15–18	0.58 (0.31–1.06)	0.58 (0.28–1.20)		0.46 (0.20–1.07)	NA
> 18	0.41 (0.19–0.91)	0.43 (0.17–1.09)		0.38 (0.13–1.11)	NA
**Time since last intercourse**					
≤ 6	1 (ref)	1 (ref)		1 (ref)	1 (ref)
> 6	2.37 (1.33–4.22)	3.09 (1.53–6.25)		2.39 (1.11–513)	10.90 (0.72-165.34)
**Condom use**					
Ever	0.51 (0.28–0.93)	0.33 (0.16–0.70)		0.30 (0.13–0.70)	0.56 (0.08–4.01)
Never	1 (ref)	1 (ref)		1 (ref)	1 (ref)
**Oral sex**					
Ever	0.76 (0.42–1.38)	0.49 (0.24–0.99)		0.56 (0.25–1.23)	0.22 (0.02–2.58)
Never	1 (ref)	1 (ref)		1 (ref)	1 (ref)

Conf: confounders

NA: Estimates were not computed because of convergence issues in the regression model due to lack of subjects in certain categories

^a^OR adjusted for age, sex, region, cigarette quantity and duration combined, alcohol quantity and level of education

^b^OR adjusted for age, sex, region, cigarette quantity and duration combined, alcohol quantity and level of education and high-risk HPV status

We studied these behaviours more closely. Age at first intercourse remained significantly associated only among persons who never used condoms. Similarly, the significant association disappeared following the adjustment on ever condom use (Table 3). Higher numbers of partners was not associated with HNSCC regardless of oral sex frequency (Table S1).

**Table 3:** Association between age at first intercourse and HNSCC stratified by ever condom use

Age at first sexual intercourse	All subjects		Condom use			
			Ever		Never	
	OR^b^	(95% CI)	OR^a^	(95% CI)	OR^a^	(95% CI)
< 15	1	ref	1	ref	1	ref
15–18	0.75	(0.40.1.45)	1.03	(0.34–3.14)	0.48	(0.14–1.60)
> 18	0.50	(0.22–1.15)	1.20	(0.46–3.14)	0.21	(0.05–0.95)

^a^OR adjusted for age, sex, region, cigarette quantity and duration combined, alcohol quantity and level of education

^b^Model from “a” further adjusted for ever condom use

We were also examined the association between Hr-HPV and HNSCC risk taking into account significant sexual behaviour variables individually and in different combinations as covariates in the multivariate model (Table S2). This helps to verify potential confounding effects between sexual behaviour variables of on HNSCC risk. The introduction of ever condom use, and/or oral sex tended to increase slightly the association between Hr-HPV and HNSCC risk (relative variation = 15–26%) contrarily to age at first sexual intercourse which caused a slight decrease.

### Oral HPV and sexual behaviour in population controls and in HNSCC cases

After adjusting for confounding factors, none of the sexual behaviour variables studied in the current paper were significantly associated with oral HPV infections in controls (Table 4).

In contrast, the associations between sexual behaviour and oral Hr-HPV infections were consistently more apparent in HNSCC cases. Cases who had a sexual debut between the ages of 15–18 years were significantly less likely to be positive for Hr-HPV. Non-heterosexuals cases were significantly more likely to have an oral Hr-HPV infection when compared to heterosexuals. Practicing oral sex regularly (often or always) was associated with Hr-HPV positivity when compared to cases who never practiced. Cases were significantly more likely to be positive for Hr-HPV when they had multiple sexual partners simultaneously from more than 5 years preceding the interview.

**Table 4:** Association between sexual behaviour and oral HPV infection in population controls and HNSCC cases

	Control					Case			
	Hr-HPV+	Hr-HPV-				Hr-HPV+	Hr-HPV-		
	*n* (col%)	*n* (col%)	OR^a^	95% CI		*n* (col%)	*n* (col%)	OR^a^	95% CI
**Age at first sexual intercourse (years)**									
< 15	7 (23.3)	45 (16.6)	1	ref		9 (50.0)	21 (31.3)	1	ref
15–18	18 (60.0)	149 (55.0)	1.05	(0.38–2.91)		7 (38.9)	36 (53.7)	0.15	(0.03–0.85)
> 18	5 (16.7)	77 (28.4)	0.65	(0.18–2.36)		2 (11.1)	10 (14.9)	0.10	(0.01–1.44)
**Time since last intercourse (months)**									
≤ 6	27 (93.1)	213 (79.5)	1	ref		11 (64.7)	34 (54.0)	1	ref
> 6	2 (6.9)	55 (20.5)	0.26	(0.05–1.27)		6 (35.3)	29 (46.0)	0.93	(0.20–4.31)
**Number of lifetime partners**									
1 to 5	8 (27.6)	124 (46.8)	1	ref		6 (35.3)	33 (51.6)	1	ref
6 to 20	15 (51.7)	106 (40.0)	1.89	(0.69–5.16)		6 (35.3)	17 (26.6)	3.88	(0.32–46.74)
> 20	6 (20.7)	35 (13.2)	1.27	(0.34–4.70)		5 (29.4)	14 (21.9)	8.59	(0.86–85.66)
**Sexual orientation**									
Heterosexual	26 (86.7)	237 (89.1)	1	ref		9 (50.0)	51 (86.7)	1	ref
Non-Heterosexual	4 (13.3)	29 (10.9)	0.78	(0.23–2.67)		9 (50.0)	12 (13.3)	6.37	(1.23–33.04)
**Condom use, frequency**									
Never	4 (13.3)	61 (22.7)	1	ref		6 (33.3)	28 (45.9)	1	ref
Once, sometimes	19 (63.3)	120 (44.6)	1.98	(0.57–6.83)		8 (44.4)	22 (36.1)	3.41	(0.57–20.22)
Often, always or almost always	7 (23.3)	88 (32.7)	0.79	(0.19–3.35)		4 (22.2)	11 (18.0)	2.10	(0.29–15.20)
**Oral sex, frequency**									
Never	4 (13.3)	73 (27.9)	1	ref		5 (27.8)	27 (42.2)	1	ref
Once, sometimes	15 (50.0)	140 (53.4)	1.52	(0.44–5.28)		5 (27.8)	25 (39.1)	0.46	(0.05–3.85)
Often, always or almost always	11 (36.7)	49 (18.7)	2.72	(0.69–10.76)		8 (44.4)	12 (18.8)	11.06	(1.12-109.06)
**Years since last oral sex**									
Never oral sex	4 (13.8)	73 (28.2)	1	ref		5 (31.3)	27 (44.3)	1	ref
< 1	19 (65.5)	105 (40.5)	2.67	(0.74–9.60)		9 (56.3)	10 (16.4)	6.69	(0.79–56.63)
1–10	5 (17.2)	57 (22.0)	1.13	(0.26–4.93)		2 (12.5)	15 (24.6)	0.29	(0.02–5.12)
> 10	1 (3.5)	24 (9.3)	0.57	(0.06–5.85)		0 (0.0)	9 (14.8)	NA	NA
**Received sperm in mouth**									
Never oral sex, never sperm, just once	12 (85.7)	164 (91.6)	1	ref		5 (71.4)	45 (90.0)	1	ref
Sometimes, often, always or almost always	2 (14.3)	15 (8.4)	9.84	(0.47-207.68)		2 (28.6)	5 (10.0)	4.91	(0.10-239.62)
**Paid for sex**									
Ever	10 (33.3)	62 (22.8)	1	ref		6 (33.3)	14 (21.5)	1	ref
Never	20 (66.7)	210 (77.2)	1.20	(0.49–2.95)		12 (66.7)	51 (78.5)	0.58	(0.13–2.62)
**Sexually transmitted infection, Frequency**									
Never	15 (51.7)	176 (65.4)	1	ref		14 (82.4)	45 (71.4)	1	ref
Once	7 (24.1)	43 (16.0)	0.63	(0.12–3.23)		0 (0.0)	6 (9.5)	NA	NA
More than once	7 (24.1)	50 (18.6)	1.82	(0.70–4.72)		3 (17.7)	12 (19.1)	0.41	(0.05–3.17)
**Recent multiple partners**									
Never multiple partners	16 (53.3)	172 (63.5)	1	ref		10 (55.6)	43 (67.2)	1	ref
≤ 5 years	3 (10.0)	27 (10.0)	0.73	(0.18–3.07)		1 (5.6)	4 (6.3)	2.18	(0.07–70.68)
> 5 years	11 (36.7)	72 (26.6)	1.15	(0.47–2.84)		7 (38.9)	17 (26.6)	6.07	(1.05–35.28)

NA: Estimates were not computed because of convergence issues in the regression model due to lack of subjects in certain categories

^a^OR adjusted for age, sex, region, cigarette quantity and duration combined, alcohol quantity and level of education

## Discussion

This is the first study addressing sexual behaviour and HNSCC in an Afro-Caribbean population. The data from our study revealed significant associations between age at first intercourse condom use, time since last sexual intercourse, and HNSCC risk. We were also interested in the association between oral Hr-HPV infection and sexual behaviour. we found no clear association among the population controls with any of the sexual behaviour variables studied. Case-to-case comparisons however, yielded evidence that is in favour of an association between risky sexual behaviour and oral Hr-HPV infection.

Oral sex and number of sexual partners [7, 14] were not associated with HPV transmission and HNSCC in our study. However, we highlighted associations with other sexual behaviour indicators. We found a significant protective association between ever condom use and HNSCC, similarly to another case-control study on oropharyngeal cancer [15].

Regarding age at first sexual intercourse, we observed a greater risk of HNSCC among subjects with sexual debut at a younger age compared to a sexual debut after the age of 18. These findings coincided with other studies [15, 16]. The observed association with age at first intercourse disappeared after adjusting for ever condom use, and in subgroup analysis among persons who used condoms regularly (often of always). On the other hand, this significant association was maintained in the subgroup of person who used condoms very inconsistently or not at all; thus, reinforcing the evidence that association between age at sexual debut and HNSCC is mediated by risky sexual habits [17].

We did not find any evidence of associations with sexual behaviour mediated by oral Hr-HPV infections. Indeed, ever condom use was significantly associated with a reduction in HNSCC risk; however, this association was attenuated neither after adjusting for Hr-HPV nor in Hr-HPV-negative subjects alone. Furthermore, the association between condom use and oral Hr-HPV in both control and cases was non-significantly negative. Contrarily to a Canadian study [14], our results allude to a risk reduction by condom use which is independent of oral Hr-HPV infections.

We studied sexual behaviour and oral Hr-HPV infections. On one hand, we did not highlight any clear association in the control group. Although non-significant, oral sex appeared to increase the risk of Hr-HPV infections in population controls which coincided with a previous study [14]. Given the absence of significant association with sexual behaviour and Hr-HPV in population controls, other factors such as fomites or self-inoculation could be considered as means of contamination in the general population [18]. On the other hand, sexual behaviour was consistently associated higher risk of oral Hr-HPV infection among our cases. We adjusted for the main confounding factors but we cannot rule out residual confounding. In particular, we did not have any data on HIV infection. In light of the risky behaviour among cases, the association with oral Hr-HPV and HNSCC may be driven by an HIV infection.

Concerning the link between sexual behaviour and HNSCC, participants who did not have sexual intercourse in the past 6 months were significantly more likely to have HNSCC. There were no studies which looked at this particular variable [8]. However, the lack of sexual intercourse from the last 6 months among cases could have arisen from bodily changes linked to their cancer. These bodily changes could reduce their desire to initiate in sexual intercourse [19]. Moreover, the presence of an STI is also a plausible hypothesis for this association with recent sexual intercourse as it has been shown to reduce sexual risk behaviour [20, 21]. In the FWI, the latter is probable because of high HIV-seropositivity [22]. HIV is known to be associated with greater HPV prevalence and can potentiate the carcinogenic activity of an HPV infection. The greater odds of Hr-HPV infection through sexual behaviour among cases might be explained by HIV induced immunodeficiency [23–28].

Our study presents several limitations. Our findings are exposed to the possibility of a recall bias due to the retrospective nature of the case-control design. Furthermore, we had a small sample size and we were not able to perform analyses by anatomical subsite. This also limited stratified analyses by Hr-HPV status. The Hr-HPV-positive subgroup was particularly small. Confidence intervals for this group were wide and in certain instances, estimates were not computed.

In addition, sexual behaviour in the Caribbean is regarded as a taboo [29] and could induce misclassification bias; in particularly, in regards to number of sexual partners. Our sample comprising mainly men, the average number of sexual partners may be more likely to be overestimated [30]. In terms of sexual orientation, homosexuality is thought to be underestimated in our sample because of discrimination faced by this group in the FWI [31]. Furthermore, the use of oral HPV detection to assess the Hr-HPV status may have caused misclassification. Oral HPV detection has been shown to have good specificity but moderate sensitivity for HPV-positive HNSCC tumours [12].

Despite these limitations in HPV detection, our study still provides valid knowledge on the association between sexual behaviour and HNSCC, and also oral HPV infection in this specific population. During our study, we did not collect any information on HIV status which is a factor suspected to modify the natural history of HPV in HNSCC [27]. The link between HNSCC and sexual behaviour could be further explained by HIV considering its high prevalence HIV in the FWI [22]. Selection bias may not be ruled out but is thought to be kept to minimum in the present analysis. There were more missing data in cases than in controls; however, we do not believe that omission of this part of the questionnaire was linked to sexual behaviour. In addition, the questions pertaining to sexual behaviour were at the end of the questionnaire, and cases tended to end the interview prior to those questions more often than controls due to fatigue. 27% of the data for HPV were missing in our sample; hence, this reduced statistical power in our analyses involving HPV. Comparison with cases from the cancer registries showed that our cases in our study had similar distribution for sex, age and cancer sites. Our controls can be considered representative of persons of the general population of similar age and sex. Indeed, a previous study has demonstrated that our method produces an unbiased sample of controls [10]. We also established the representativeness of our population controls after comparing our observed distribution for tobacco, alcohol and sexual behaviour with that of a national health survey [3] and a regional survey [32].

Sexual behaviour is a modifiable risk factor; therefore, there may be additional opportunities to prevent HNSCC in the FWI [33] in light of our new results. A greater understanding of biological mechanisms between HIV and other viral agents in head and neck cancer could inform clinical practice and cancer control policy. These new data can also apply to other Afro-Caribbean populations for which information on this topic is scarce.

## Conclusion

This is the first study to investigate the role of sexual behaviour in the occurrence of HNSCC in an Afro-Caribbean population while taking into account oral HPV infections. Despite our study limitations, first intercourse after 18 years, short time intervals since last intercourse and never condom use were inversely associated with HNSCC independently of oral Hr-HPV infection. Oral Hr-HPV infections were associated with riskier sexual behaviour in HNSCC cases but not in population controls. Other sources of contamination such as fomites, as well as HIV infections could play a role in the causal pathway to HNSCC. Further investigation on this topic in the FWI is warranted and special attention should be given to the interaction between viral factors to better substantiate the natural history of HPV in HNSCC thus, providing additional prospects for prevention.

## Electronic supplementary material

Below is the link to the electronic supplementary material.

**Additional file 1:** Sex behaviour HNC_BMC. **Table S1:** Number of lifetime sexual partners stratified by oral sex frequency. **Table S2:** The effect of Hr-HPV on HNSCC risk after adjusting for age at first intercourse, condom use and oral sex

## Abbreviations

CI: Confidence interval
FWI: French West Indies
HNSCC: Head and neck squamous cell carcinoma
HPV: Human papillomavirus
Hr-HPV: High-risk Human papillomavirus
OR: Odds ratio

## Declarations

### Data sharing statement

The datasets generated and/or analysed during the current study are not publicly available since personal health data underlying the findings are protected by the French Data Protection Act. Data are however are available from the corresponding author on reasonable request.
