# Supplementary material for: Association between sexual behaviour and head and neck cancer in the French West Indies: a case-control study based on an Afro-Caribbean population
Source: BMC Cancer. 2023 May 5;23:407. doi: 10.1186/s12885-023-10870-x (PMC10163737; doi:10.1186/s12885-023-10870-x)
Supplement: Supplementary file 1 — Additional file 1: Sex behaviour HNC_BMC. Table S1: Number of lifetime sexual partners stratified by oral sex frequency. Table S2: The effect of Hr-HPV on HNSCC risk after adjusting for age at first intercourse, condom use and oral sex [file 12885_2023_10870_MOESM1_ESM.pdf]

Title: Association between sexual behaviour and head and neck cancer in the French West Indies: a case-control study

Authors:

Aviane Auguste, Stanie Gaete, Leah Michineau, Cécile Herrmann-Storck, Clarisse Joachim, Suzy Duflo, Jacqueline Deloumeaux, Danièle Luce.

ADDITIONAL FILE

Additional file 1_Sex behaviour HNC_BMC

File type: PDF

Title and description of data:

Table S1: Number of lifetime sexual partners stratified by oral sex frequency.

Table S2: The effect of Hr-HPV on HNSCC risk after adjusting for age at first intercourse, condom use and oral sex.

Table S1: Number of lifetime sexual partners stratified by oral sex frequency.

Number of lifetime partners	Oral sex frequency			
	Often or always		Never, Once, Sometimes	
	OR	95% CI	OR	95% CI
1 to 5	1.00	ref	1.00	ref
6 to 20	0.34	(0.06-1.88)	0.52	(0.25-1.09)
> 20	0.62	(0.12-3.22)	0.68	(0.26-1.78)

a: OR adjusted for age, sex, recruitment site, cigarette quantity and duration combined, alcohol quantity and level of education.

Table created by the authors

Table S2: The effect of Hr-HPV on HNSCC risk after adjusting for age at first intercourse, condom use and oral sex.

	OR	(95% CI)
Hr-HPV-	1	ref
**Covariate(s)**		
Univariate[a]	2.23	(1.17-4.25)
Univariate+Confounders[b]	2.23	(0.98-5.11)
Univariate+Confounders[b]+:		
Age at first sexual intercourse	2.00	(0.86-4.70)
Condom use	2.69	(1.13-6.39)
Oral sex	2.57	(1.11-5.96)
Condom use+ Oral sex	2.80	(1.18-6.64)
Age at first sexual intercourse+Condom use	2.46	(1.02-5.93)
Age at first sexual intercourse+Oral sex	2.41	(1.02-5.67)
Age at first sexual intercourse+Condom use+ Oral sex	2.59	(1.08-6.23)

a: OR adjusted for age, sex, recruitment site
b: OR adjusted for age, sex, recruitment site, cigarette quantity and duration combined, alcohol quantity and level of education
Table created by the authors
